# Associations between Bovine β-Defensin 4 Genotypes and Production Traits of Polish Holstein-Friesian Dairy Cattle

**DOI:** 10.3390/ani9100723

**Published:** 2019-09-25

**Authors:** Paulina Brodowska, Lech Zwierzchowski, Sylwester Marczak, Wiesław Jarmuż, Emilia Bagnicka

**Affiliations:** Institute of Genetics and Animal Breeding, Polish Academy of Sciences, Postepu 36A, 05-552 Jastrzębiec, Poland; p.brodowska@ighz.pl (P.B.); l.zwierzchowski@ighz.pl (L.Z.); s.marczak@ighz.pl (S.M.); wieslaw.jarmuz@gmail.com (W.J.)

**Keywords:** dairy cattle, production trait, mastitis, defensin, single nucleotide polymorphism, association

## Abstract

**Simple Summary:**

Mastitis negatively affects dairy cattle, causing inferior milk quality and premature animal culling, which leads to economic losses. Therefore, selection based on genetic markers (i.g., marker-assisted selection) should also include functional traits with low heritability, such as resistance to udder inflammation. Single nucleotide polymorphisms (SNPs) identified in genes involved in the immune system, such as defensins with antibacterial properties, could be valuable markers. We chose two SNPs within the bovine neutrophil beta-defensin 4 (*BNBD4)* gene analyzed in a previous study related to milk production and udder health. Since these SNPs are located very close to each other in the gene intron, it is useful to analyze their association with production traits as a combined genotype. The results showed that these genotypes are indeed associated with productivity, as well as functional traits (milk, fat, and protein yields, fat, protein, lactose, and dry matter contents, and somatic cell count). The differences between the results based on the phenotypic data and the breeding values of studied traits may confirm the results of simulation studies that indicate a high rate of false-positives in genome-wide association study (GWAS) based on classically calculated estimated breeding values (EBVs) using best linear unbiased prediction (BLUP) methodology.

**Abstract:**

This study analyzed the associations between two single-nucleotide polymorphisms (C2239T and A1674C), used together as a genotype located in *BNBD4,* and milk traits and breeding values of productivity traits of Polish Holstein-Friesian dairy cows. The research was carried out on 322 cows, with 7070 milk parameter and somatic cell count records in daily milking, as well as 897 records covering data on whole lactations, and 2209 breeding value records for productivity traits. The DMU statistical package with a one-trait repeatability test-day animal model was used to estimate the associations. The differences between the genotype effects were analyzed using Duncan’s post-hoc tests. The CC/AA and CT/AC genotypes had the highest frequencies (0.62 and 0.23, respectively). For use in marker-assisted selection, the CC/AC genotype is the most promising as an indicator of high-yielding cows potentially resistant to mastitis, because it was associated with the lowest somatic cell count (SCC), highest milk, fat, and protein yields in daily milking, as well as with milk yield in the whole lactation. The studied genotypes were also related to the breeding values of all the investigated production traits. However, some simulation studies have indicated a high rate of false-positives in GWAS based on classically calculated EBVs.

## 1. Introduction

Mastitis affects dairy cattle populations worldwide, especially those in intensive production conditions, causing economic losses and reduced milk quality [1,2]. The resistance of the cows to mammary gland inflammation is a functional trait with a low heritability coefficient (0.01–0.15) [3]. Thus, it is necessary to find reliable and effective markers for selection to genetically improve the resistance to mastitis in high-yielding dairy cows [4]. Searching for the genetic polymorphisms that may be associated with functional or production traits in dairy cattle has been the aim of numerous studies [5,6,7,8]. Single nucleotide polymorphisms (SNPs), as genetic markers of functional or production traits, are a very helpful tool for estimating the genetic value of animals early in life to achieve desirable traits genetically, when they are in linkage disequilibrium and they phase with the genetic variation [9]. Quantitative trait loci (QTL)-linked SNP markers and marker-assisted selection (MAS) are considered part of modern, profitable breeding programs [10]. Predicting the exact genetic breeding values using dense SNP marker maps has changed genetic improvement strategies [11]. For example, after a simulation of economic analysis of the implementation of the cows’ genomic assessment at the dairy farm level, researchers noticed significant advantages, such as a doubling of the genetic progress and a 92% reduction in the bulls’ evaluation costs [12]. In MAS programs, genetic progress is accelerated mainly by increasing the accuracy of selection and shortening the generation interval. Therefore, MAS is an especially useful tool for selecting traits for which conventional methods do not accurately estimate the breeding value, e.g., traits with low heritability, recessive traits, and traits that are difficult and expensive to determine [13].

High-performance genotyping techniques are now available for high-density SNP systems [14]; however, until now, only a small subset of SNPs has been developed into genetic markers for genomic selection, linkage disequilibrium models, or genome-wide association studies [15]. Despite the discovery of a large number of SNPs and production of QTL maps, most have a very small effect on quantitative traits. Therefore, it is still necessary to search for specific SNPs, which should have a significant impact on desirable traits [16].

Some interesting genetic marker candidates for mastitis resistance may be the polymorphisms of genes encoding bovine defensins, which belong to a large, ancient, and varied group of antimicrobial peptides [17]. The defensins, as a class of antimicrobial peptides, were characterized in some papers [18,19,20,21]. Briefly, defensins are found in plants, invertebrates, and vertebrates. They comprise three classes: Alpha, beta, and theta; however, class alpha was found only in primates, lagomorphs, and rodents and only in several species of other mammals (horse, African elephant, and *Didelphimorphia* genus), until recently. The theta defensins were found only in rhesus monkeys. Thus, the genes of the beta class seem to be the most common ones. It is suggested that defensin genes evolved over 130 million years before divergence of placental mammals from marsupials; however, other researchers imply that the common defensin genes existed even before reptiles and *aves* diverged. The alpha- and beta-defensins form beta-sheet structures that are intramolecularly stabilized by disulfide bonds between cysteine residues.

β-Defensins have many functions, including antimicrobial activity against microorganisms: Gram-positive and Gram-negative bacteria, enveloped viruses, and fungi that cause, e.g., mastitis [22]. A cluster of defensin genes was found on bovine chromosome 27 [23], on which QTLs affecting body structure and dairy traits were also identified [24]. Moreover, Kościuczuk et al. [25] found their increased expression in mammary gland tissues infected with both coagulase-positive and coagulase-negative staphylococci. A few studies on the polymorphisms of defensin genes in dairy cows have been conducted [21,26,27,28], though the bovine neutrophil beta-defensin 4 (*BNBD4)* gene polymorphism has been analyzed more accurately [17,29,30]. In those studies, two polymorphisms of the *BNBD4* gene (GenBank no. AF008307) were identified and analyzed separately: C→T at position 2239 using *Nla*III restriction enzyme and A→C at position 1674 using *Bsr*I restriction enzyme. Both loci are located in the intron of the *BNBD4* gene (the bovine *BNBD4* gene (450 bp) consists of two exons (193 and 153 bp, respectively) and one intron (1485 bp)). The *Nla*III C2239T polymorphism was associated with protein, fat, and lactose content, in addition to somatic cell count in daily milk as well as during the whole lactation period. Cows carrying the CC genotype of *Nla*III produced milk with a higher fat and lactose content and a lower milk somatic cell count (SCC), thus they could be more resistant to mastitis. The CT genotype at position 2239 was also associated with the highest milk yield and protein content [17]. The *BsrI* A1674C polymorphism also showed a relationship with milk yield and composition. Cows carrying the AA genotype had the highest levels of daily milk and fat yields, as well as energy-corrected milk (ECM) during the whole lactation period. Cows carrying the CC genotype had the highest protein and fat contents in their milk, while cows with the AC genotype were associated with the highest daily fat yield and ECM and the lowest SCC in milk based on monthly recording [31]. The genotypes described above, studied separately, seem to be associated with production traits, but may also be indicators of the health state of the mammary gland.

Therefore, the aim of this study was to continue the analysis of the association between *BNBD4* gene polymorphisms and production/functional traits, but as a combination of the two identified SNPs—*Nla*III C2239T and *Bsr*I A1674C. Since these SNPs are located very close to each other in the gene (less than 600 bp apart), it is highly probable that they are inherited together. We analyzed the associations between the *BNBD4* genotype of the two SNPs (*Nla*III C2239T and *Bsr*I A1674C) and the animals’ production and functional traits (traits in daily milking: Milk, fat, protein, and energy corrected milk (ECM) yields, and fat, protein, lactose, and dry matter contents, and somatic cell count; in both whole milk, ECM, fat, and protein yields, and fat and protein contents, as well as the breeding value of the milk, fat, and protein yields, and fat and protein contents).

## 2. Materials and Methods

### 2.1. Animals

The study was carried out on 322 Polish Holstein-Friesian dairy cows of the Black and White variety, maintained in a loose housing system at a dairy cattle farm in Central Poland. The cows were between their first and eighth lactations and were the daughters of 116 bulls. The pedigree file included information on 1564 animals from three generations.

The tested animals were fed a complete total mixed ration (TMR) of corn silage (75%), concentrates (20%), and hay (5%), supplemented with minerals and vitamins according to a system developed by the Institut National de la Recherche Agronomique (INRA) of France and adopted by the Research Institute of Animal Production (IZ PIB), Poland [32]. The animals had unlimited access to hay and water. The average annual milk yield amounted to ~9000 kg of milk, containing 4.0% fat and 3.4% protein on average. Cows were milked mechanically twice a day using DeLaval equipment in a fishbone milking parlor (Sweden).

The information on the genotyped cows was previously presented by Bagnicka and co-workers [17,30]. Briefly, a polymorphism of the *BNBD4* gene was found: A C→T transition at position 2239 detected by *Nla*III restriction enzyme and an A→C transversion at position 1674 detected by the *Brs*I enzyme. Both SNPs were combined into defensin genotypes (DGs). The daily milk yield, the fat, protein, lactose, and dry matter contents, and the SCC were measured by an independent laboratory belonging to the Polish Federation of Cattle Breeders and Dairy Farmers—the only organization authorized by Ministry of Agriculture and Rural Development to keep herd book for dairy cattle, provide milk recording and type evaluation in Poland. This information was used in test-day model analysis. Phenotypic data regarding the milk, fat, and protein yield and content from the whole lactation were also collected. Moreover, estimations of the breeding values of the traits and pedigree information, extracted from the Polish national dataset from the Polish Federation of Cattle Breeders and Dairy Farmers, were also gathered.

A total of 7070 records on daily milk yield and milk composition (phenotypic data) were collected. There were 897 records covering information on milk, fat, and protein yield, as well as fat and protein contents during whole lactations (phenotypic data). Records with lactation periods shorter than 270 days were omitted. The longest whole lactation period lasted 749 days. Moreover, 2209 records about the estimated breeding values (EBVs) of five investigated traits (milk, fat, and protein yield, and fat and protein contents) obtained using “classic” BLUP (best linear unbiased prediction) methodology, in total, were available. The EBVs for cows in the active Polish population are calculated in National Institute of Animal Production in Krakow three times each year using both BLUP and genomic best linear unbiased prediction (GBLUP) methods.

### 2.2. Statistical Analysis

To determine the association between the identified polymorphisms and the daily production traits, a one-trait repeatability test-day animal model with the DMU package was used [32]. The model included the dates of tests, year/season of calving, parity, and defensins’ genotype as fixed effects, as well as an animal additive genetic effect and permanent environmental effect as random effects. The effect of the stages of lactation was modeled using Legendre polynomials nested within parity [33] up to the 5th power. In the second analysis, to determine the association between DGs and lactation milk traits, again, the one-trait repeatability animal model with the DMU package was used, where animal additive genetic effects and permanent environmental effects were designated as random, and genotype, parity, and year/season of calving were designated as fixed effects. Next, in the third analysis, to estimate the relationship between the DGs of the β4-defensin gene and the EBVs of production traits, a one-trait repeatability animal model was used. The model included additive animal genetic effects and permanent environmental effects as random effects and the defensins’ genotype and age of estimation of breeding value of production traits (each animal had more than one EBV estimated at different age) as fixed effect. The ECM value was established according to the formula: ECM (kg/day) = milk (kg/day) × [38.3 × fat (g/kg) + 24.2 × protein (g/kg) + 16.54 × lactose (g/kg) +20.7]/3.140 [34].

SCC value was transformed to the natural logarithmic scale (lnSCC) before the statistical analysis. The differences between the DG effects were analyzed using Duncan’s post-hoc test. The differences between the observed and expected frequencies of the genotypes were estimated using the χ^2^ test. Moreover, the D’ (coefficient of linkage disequilibrium, which measures the level of linkage disequilibrium) and r^2^ (the square of the correlation coefficient between two SNPs to predict the allele at the second *locus* based on information on the allele in one *locus*) statistics were calculated, which are two of the most commonly used measures for extent of Linkage Disequilibrium (LD), using CubeX software (Developer: The Bristol Genetic Epidemiology Laboratories, Bristol, UK) [35].

## 3. Results

Both SNPs were combined into six DGs: CC/AA, CC/AC, CC/CC, CT/AA, CT/AC, and CT/CC, with frequencies of 0.62, 0.08, 0.02, 0.03, 0.23, and 0.02, respectively. The most common DGs were the double homozygous CC/AA and the double heterozygous CT/AC. Three possible genotypes, TT/CC, TT/AC, and TT/AA, were not present in the animals included in this study. The observed genotype frequencies differ (*p* < 0.01) from expected ones, which means that the population is not in Hardy–Weinberg proportions. The D’ statistic was obtained as 0.85 (the two SNPs are co-inherited in approximately 85% of cases) and r^2^ was equal to 0.5, which both indicate the non-random association between *loci*.

The CC/AC genotype was associated with the highest milk, fat, protein, and lactose contents and also the lowest lnSCC in daily tests (Table 1). However, the milk yield associated with this DG was only average and ranged between 30.72 and 24.44 kg/day. The lowest milk, fat, and protein yields and lactose content were associated with the CT/CC genotype, which was also the genotype with the lowest frequency within the studied group. However, this DG was connected with the highest fat, protein, and dry matter contents. The lowest lactose content was connected with the CC/CC and CT/CC genotypes, while the lowest fat and dry matter contents were associated with the double heterozygous CT/AC genotype. The highest lnSCC was associated with the CT/AA genotype. The CC/AC genotype was connected with the highest ECM value and the CT/CC genotype with the lowest; however, the CT/CC genotype had the highest percentage of dry matter. 

Associations of the DGs with milk yield, fat, and protein contents in the whole lactation were found (Table 2). During the whole lactation, the CC/AC genotype was connected with high milk and protein yield, as well as with high ECM. The lowest daily milk and protein yields were characteristic of the CC/CC genotype, while the lowest ECM was found for the CT/AA genotype. The CC/AA genotype was connected with the highest fat yield but also with the lowest protein content. Cows with the CC/CC genotype had the highest fat content, while the lowest fat content was found in cows with the CC/AA and CT/AC genotypes.

The CT/AA genotype was connected with the highest breeding value of all the investigated traits, but unfortunately, its frequency was very low within the studied group (Table 3). Some differences were found between the results obtained for the phenotypic values of the investigated traits and their breeding values. However, the CT/CC genotype was characterized by the lowest milk, fat, and protein yield, both in phenotypic and breeding values, and these results are consistent with those obtained from the phenotypic data based on daily milking. This DG was characterized by very low frequency, which may mean that cows carrying the CT/CC genotype were eliminated from breeding.

Summing up, the CC/AC genotype was connected with very high phenotypic values for the most traits, while the CT/AA genotype was associated with the highest breeding values of all investigated traits.

## 4. Discussion

It is recognized that antimicrobial peptides are one of the oldest defense weapons of living organisms [36]. In a study of the expression levels of β-defensin genes in dairy cows, much higher expression levels of these genes were found in mammary gland parenchyma derived from udder quarters infected with coagulase-positive or coagulase-negative staphylococci than in infection-free secretory tissue [25]. Moreover, Meredith et al. [37] and Tetens et al. [38] stressed that the cluster of defensin genes is a candidate QTL region affecting the number of somatic cells in milk. As mentioned above, polymorphisms of β-defensin genes have been the subject of much research; however, most of these studies concerned the role of human defensins in human health [39,40,41]. Some variants of these genes could be useful as markers of the risk of diseases in humans, such as chronic obstructive pulmonary disease [42] and asthma [43], and have also been connected with protective effects against oral candidiasis [39] and the risk of HIV-1 infection [44,45].

According to the present and earlier studies [17,29], there is a strong association between polymorphic forms of the *BNBD4* gene and the SCC, which is an indicator of the health state of the mammary gland. The health status of the udder influences both the yield and chemical composition of milk; thus, it has a substantial impact on the technological parameters of milk [46]. By ensuring their welfare, animals with healthy udders can be used for a long time without harm. Since defensins protect the mammary gland from bacterial, viral, or fungal infections, the polymorphic forms of their genes could be potential genetic markers for an animal’s susceptibility or resistance to mastitis.

In this study, we found with a high probability that polymorphisms of the *BNBD4* gene are associated with functional and production traits in dairy cattle. The differences between the results obtained for the phenotypic and breeding values (estimated using classical method BLUP) of the studied traits may confirm the results of simulation studies that indicate a high rate of false-positive results in genome-wide association study (GWAS) based on classically calculated EBVs [47]. However, further studies in another dairy cattle population are necessary. Such research is needed because large amounts of phenotypic data are necessary to estimate the effects of genotypes on production and functional traits with enough accuracy [48]. Thus, studies on large dairy cattle populations are required before markers for the *BNBD4* gene are recommended for inclusion in selection programs. 

## 5. Conclusions

In this research, we were able to determine some associations between polymorphisms of *BNBD4* genotypes and milk traits, as well as some indicators of mammary gland health state, e.g., SCC, and lactose and total protein contents. Based on the daily milking data, many relationships were found between traits and genotypes. However, during the analysis of the whole lactation data, these relationships seemed to be much weaker, probably due to both the many conversions of raw data, which increase the information biases, and to the low amount of data. That being said, from our study, we can conclude that a calf carrying the CT/CC genotype should not be bred, while those carrying the CC/AC or CT/AA combined genotypes should be used as replacements in the herd. However, because of the very low frequencies of all beneficial genotypes, further studies on much larger populations are needed.

## Figures and Tables

**Table 1:** The estimates and their standard errors (SE) of defensin genotypes for the investigated traits in daily tests.

Genotype	*N*	Estimate (SE)								
		Milk kg	ECM	Fat kg	Protein kg	Fat %	Protein %	Lactose %	lnSCC	Dry Matter %
**CC/AA**	4761	29.49 **^A^** (1.15)	31.01 **^A^** (1.17)	1.25 **^A^** (0.08)	0.35 **^A^** (0.04)	4.39 **^A,a^** (0.14)	3.55 **^A^** (0.06)	4.54 **^A^** (0.05)	7.25 **^a^** (0.23)	13.23 **^A^** (0.17)
**CC/AC**	625	30.72 **^B^** (1.44)	32.66 **^B^** (1.42)	1.32 **^B^** (0.09)	0.39 **^B^** (0.05)	4.42 **^a^** (0.17)	3.55 **^A^** (0.07)	4.57 **^A^** (0.06)	7.11 **^A^** (0.33)	13.31 **^a^** (0.21)
**CC/CC**	163	29.20 **^A^** (2.00)	31.91 **^A^** (1.92)	1.31 **^B^** (0.10)	0.34 **^A^** (0.06)	4.56 **^B^** (0.23)	3.56 **^A^** (0.09)	4.46 **^B,a^** (0.08)	7.18 **^a^** (0.51)	13.34 (0.30)
**CT/AA**	145	30.09 **^A,B^** (1.82)	30.00 **^A^** (1.77)	1.21 **^A^** (0.09)	0.36 **^A^** (0.05)	4.44 (0.22)	3.56 **^A^** (0.08)	4.49 **^B^** (0.07)	7.47 **^B,b^** (0.46)	13.31 (0.27)
**CT/AC**	1875	29.38 **^A^** (1.22)	30.72 **^A^** (1.23)	1.24 **^A^** (0.08)	0.36 **^A^** (0.04)	4.36 **^A,a^** (0.16)	3.56 **^A^** (0.06)	4.52 **^B,b^** (0.05)	7.28 (0.25)	13.19 **^A,b^** (0.19)
**CT/CC**	61	24.44 **^C^** (2.64)	25.04 **^C^** (2.54)	1.04 **^C^** (0.12)	0.27 **^C^** (0.07)	4.61 **^b^** (0.32)	3.74 **^B^** (0.12)	4.46 **^B,a^** (0.10)	7.37 (0.70)	13.54 **^B^** (0.41)

*N*—number of observations. SE—standard error. A, B, C, D, E, F—different letters indicate differences at *p* ≤ 0.01. a, b—different letters indicate differences at *p* ≤ 0.05. ECM—energy-corrected milk yield. lnSCC—somatic cell count transformed into natural logarithmic scale.

**Table 2:** The estimates and their standard errors (SE) of the effects of defensin genotypes for the investigated traits during the whole lactation.

Genotype	*N*	Estimate (SE)					
		Milk kg	ECM kg	Fat kg	Protein kg	Fat %	Protein %
**CC/AA**	382	10,994.14 **^a^** (395.72)	12,235 **^a^** (503)	423.58 (14.58)	329.93 (17.07)	3.90 **^A^** (0.11)	3.25 **^a^** (0.05)
**CC/AC**	57	11,379.85 **^b^** (482.32)	12,682 **^b^** (571)	362.02 (17.33)	340.44 (14.74)	3.93 **^a^** (0.14)	3.27 (0.06)
**CC/CC**	14	10,487.42 **^a^** (704.34)	1231 (752)	379.35 (24.66)	317.39 (21.32)	4.17 **^B,b^** (0.21)	3.28 (0.09)
**CT/AA**	12	10,487.94 **^a^** (710.65)	11,428 **^a^** (765)	379.93 (25.24)	317.78 (22.46)	3.91 (0.21)	3.31 **^b^** (0.09)
**CT/AC**	151	10,895.82 **^a^** (421.97)	12,076 **^a^** (528)	370.04 (15.35)	339.70 (13.08)	3.90 **^A^** (0.12)	3.28 (0.05)
**CT/CC**	13	10,355.52 **^a^** (714.44)	11,594 **^a^** (764)	379.88 (25.19)	324.94 (22.61)	4.06 (0.21)	3.31 **^b^** (0.09)

*N*—number of observations. SE—standard error. A, B—different letters indicate differences at *p* ≤ 0.01. a, b—different letters indicate differences at *p* ≤ 0.05. ECM—energy-corrected milk yield.

**Table 3:** The estimates and their standard errors (SE) of effects of defensin genotypes for the breeding values of the investigated traits.

Genotype	*N*	Estimate (SE)				
		Milk kg	Fat kg	Protein kg	Fat %	Protein %
**CC/AA**	1278	73.47 **^A^** (114.00)	−11.29 **^A^** (4.78)	−7.79 **^A^** (3.71)	−0.08 **^A^** (0.03)	−0.04 **^A^** (0.01)
**CC/AC**	182	156.67 **^B^** (129.53)	−8.44 **^B,b^** (5.22)	−5.50 **^B^** (4.09)	−0.09 **^B^** (0.05)	−0.04 **^A^** (0.02)
**CC/CC**	40	162.23 **^B^** (165.20)	−7.09 **^B^** (6.32)	−7.69 **^C^** (5.02)	−0.05 **^C^** (0.07)	−0.07 **^B^** (0.03)
**CT/AA**	37	178.16 **^B^** (170.57)	−5.82 **^B,b^** (6.47)	−2.69 **^D^** (5.16)	−0.04 **^D^** (0.08)	−0.003 **^C^** (0.03)
**CT/AC**	491	41.94 **^A^** (119.23)	−13.79 **^C^** (4.92)	−9.17 **^E^** (3.83)	−0.10 **^E^** (0.04)	−0.04 **^A^** (0.02)
**CT/CC**	10	−264.03 **^C^** (250.70)	−29.24 **^D^** (9.01)	−17.50 **^F^** (7.30)	−0.18 **^F^** (0.13)	−0.03 **^A^** (0.06)

*N*—number of observations. SE—standard error. A, B, C, D, E, F—different letters indicate differences at *p* ≤ 0.01. a, b—different letters indicate differences at *p* ≤ 0.05.
